# New insights into controlling radical migration pathways in heme enzymes gained from the study of a dye-decolorising peroxidase†

**DOI:** 10.1039/d3sc04453j

**Published:** 2023-10-06

**Authors:** Marina Lučić, Michael T. Wilson, Jacob Pullin, Michael A. Hough, Dimitri A. Svistunenko, Jonathan A. R. Worrall

**Affiliations:** a School of Life Sciences, University of Essex Wivenhoe Park Colchester Essex CO4 3SQ UK jworrall@essex.ac.uk svist@essex.ac.uk; b Diamond Light Source, Harwell Science and Innovation Campus Didcot Oxfordshire OX11 0DE UK

## Abstract

In heme enzymes, such as members of the dye-decolorising peroxidase (DyP) family, the formation of the highly oxidising catalytic Fe(iv)-oxo intermediates following reaction with hydrogen peroxide can lead to free radical migration (hole hopping) from the heme to form cationic tyrosine and/or tryptophan radicals. These species are highly oxidising (∼1 V *vs.* NHE) and under certain circumstances can catalyse the oxidation of organic substrates. Factors that govern which specific tyrosine or tryptophan the free radical migrates to in heme enzymes are not well understood, although in the case of tyrosyl radical formation the nearby proximity of a proton acceptor is a recognised facilitating factor. By using an A-type member of the DyP family (DtpAa) as an exemplar, we combine protein engineering, X-ray crystallography, hole-hopping calculations, EPR spectroscopy and kinetic modelling to provide compelling new insights into the control of radical migration pathways following reaction of the heme with hydrogen peroxide. We demonstrate that the presence of a tryptophan/tyrosine dyad motif displaying a T-shaped orientation of aromatic rings on the proximal side of the heme dominates the radical migration landscape in wild-type DtpAa and continues to do so following the rational engineering into DtpAa of a previously identified radical migration pathway in an A-type homolog on the distal side of the heme. Only on disrupting the proximal dyad, through removal of an oxygen atom, does the radical migration pathway then switch to the engineered distal pathway to form the desired tyrosyl radical. Implications for protein design and biocatalysis are discussed.

## Abstract 2

We show that radical migration in a heme peroxidase can be controlled and directed to a rationally designed site through simply removing an oxygen atom form the protein structure.

## Graphical abstract

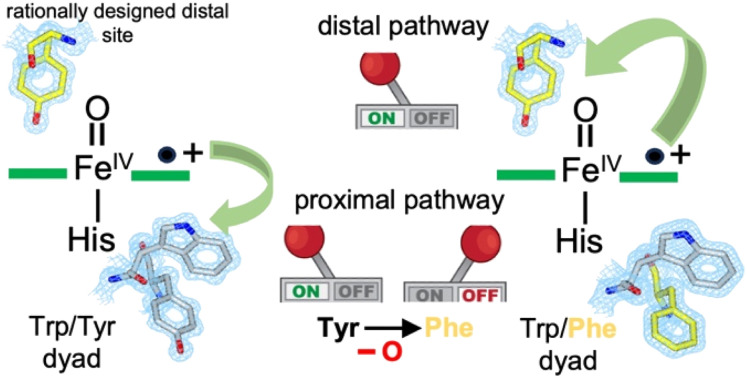

## Introduction

Heme enzymes, such as cytochrome P450s and peroxidases can react with molecular oxygen (O_2_) or hydrogen peroxide (H_2_O_2_) to generate high-valent Fe(iv)=O heme species, possessing redox potentials in the 0.75–1.35 V range, that act as potent biological oxidants.^1–5^ In the absence of a substrate, the strongly oxidising equivalents, stored on the high-potential heme, can be rapidly dissipated to nearby oxidisable amino acids *e.g.* Tyr, Trp, Met, and Cys, with the effect of potentially causing oxidative damage to the protein matrix.^6^ In X-ray crystal structures of cytochrome P450s, clusters of closely spaced Tyr and Trp residues (<10 Å apart) have been identified that can harbour a transient cation radical, termed a hole.^7^ Electron-transfer between these aromatic residues, equivalent to free radical movement, “hole hopping”, results in positive charge propagation away from the heme to the lowest potential site, often on the protein surface, where the hole can be quenched by a reductant (substrate).^7–9^ Such chains of Tyr and Trp residues, have also been identified in copper oxidases and oxygenases, and are now widely considered to serve as a protective mechanism (escape routes), preventing oxidative damage and preserving the catalytic integrity of the enzyme for when a substrate is present.^10^ In yeast cytochrome *c* peroxidase (CcP) hole hopping pathways operate to help protect, under high H_2_O_2_ levels, from irreversible oxidation of the distal His-heme ligand, which results in loss of the heme and formation of the apo-protein.^11,12^ Furthermore, hole hopping pathways can have functional implications. For example, the formation of stable, residue specific radical sites, can serve as a functional adaptation in the enzyme, through enhancing an electron-transfer pathway for substrate oxidation as is the case for the Trp π-radical cation, Trp191˙^+^ in CcP,^13–16^ or be directly involved in the oxidation of a substrate through formation of neutral surface Tyr or Trp radicals in lignin and versatile peroxidases (LiP and VP, respectively).^17–20^ Hole hopping through a series of Trp residues within a protein complex between the di-heme protein MauG and preMADH, has also been demonstrated to fulfil a functional catalytic role in this system.^21,22^

Dye-decolourising peroxidases (DyPs) are a distinct structural family of peroxidases possessing an α + β dimeric fold that houses a b-type heme coordinated on its proximal side by a His residue.^23–27^ Their name is derived from the ability of some, but not all, to oxidise recalcitrant high-potential anthraquinone and/or azo-dyes often used in the textile industry. These dyes are too bulky to access the sterically restricted heme site, and several studies have put forward mechanisms of substrate oxidation based on the observation of surface Tyr and/or Trp radicals.^28–33^ These site specific radicals are likely formed as a result of the existence of functional hole hopping pathways that move the radical from the Fe(iv)-oxo porphyrin π-cation radical compound I species, formed upon reaction of the ferric state with H_2_O_2_. The soil dwelling bacterium, *Streptomyces lividans* possesses three DyPs; two belonging to the A-type and a third belonging to the B-type sub-families.^27^ The two A-type homologs, named DtpA and DtpAa react in their ferric heme state with H_2_O_2_ to form a two-electron oxidised derivative that is an Fe(iv)=O heme carrying a porphyrin π-cation radical (Fe(iv)=O[Por˙^+^]) – termed compound I.^34–36^ In the presence of a substrate, reduction of the porphyrin π-cation radical results in the formation of compound II (Fe(iv)=O), with a second substrate molecule reducing compound II to the ferric state. With small substrates such as ferrocyanide fast direct access to the high-valent heme species is possible,^34,37^ whereas with large substrates, such as dye molecules, that cannot access the heme pocket, reduction of the high-valent heme species is generally slower and occurs through long-range electron-transfer.^30,31,35,38^ We have previously reported for DtpA, that in the absence of a substrate, the porphyrin π-cation radical migrates, and a stable neutral tyrosyl radical (YO˙) is formed, as identified by Electron Paramagnetic Resonance (EPR) spectroscopy.^35^

Oxidation of a Tyr (redox potential 1.22–0.93 V between pH 2–7)^39^ results first in the formation of the highly reactive radical cation (YOH˙^+^). Such species are rarely detected,^40^ due to a dramatic drop in p*K*_a_ from 10 to −2,^41^ leading to rapid deprotonation, and formation of a less reactive (lower redox potential) YO˙ species. The positioning of a proton acceptor (base), that can rapidly accept the proton from YOH˙^+^ and donate a hydrogen-bond (H-bond) back to the YO˙ species, results in an upshift in the redox potential of the H-bonded YO˙ species by as much as 200 mV.^42,43^ In contrast, Trp oxidation (redox potential 1.15–1.015 V between pH 2–7)^39^ results first in the formation of a TrpNH˙^+^ where the p*K*_a_ of the indole N proton is ∼4.5, much higher than a proton in water (p*K*_a_ of H_3_O^+^ ∼0) and therefore deprotonation is thermodynamically less favoured.^44,45^

**Fig. 1:**
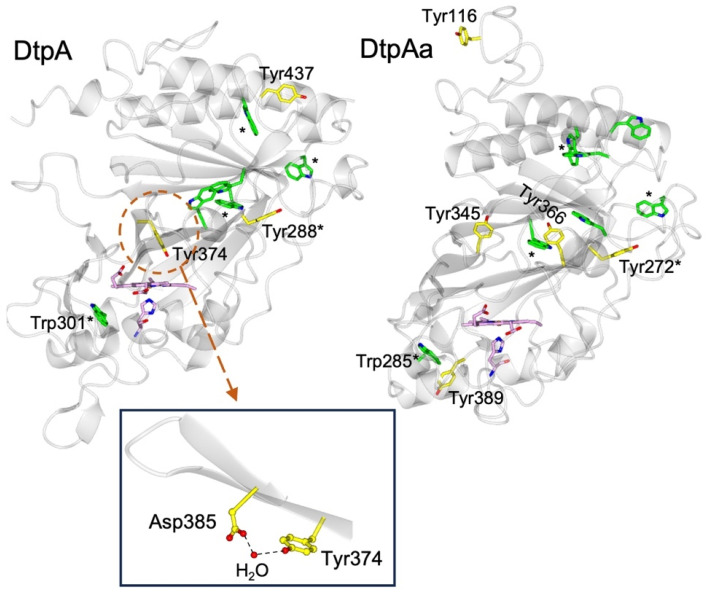
Cartoon representation of the X-ray crystal structures of ferric DtpA (PDB code 6GZW) and DtpAa (PDB code 6I43) indicating the distribution of electron-transfer active Tyr (yellow sticks) and Trp (green sticks) residues. Tyr and Trp locations that are structurally conserved between the two A-type homologues are indicated with a *. The heme and proximal His ligand are also represented in sticks and coloured purple. Inset, the hydrogen bonding interactions between Asp385, a structural H_2_O molecule and Tyr374 in DtpA.

DtpA contains 3 Tyr residues and 6 Trp residues (Fig. 1). Only Tyr374 participates in a H-bonding network, involving a structured H_2_O molecule that bridges between the phenol group and the carboxylate group of Asp385 (inset Fig. 1).^35^ Such an environment would facilitate rapid proton transfer and indeed Tyr374 was identified through EPR spectroscopy to be the location of a stable YO˙ species.^35^ Although Tyr374 is not surface exposed (unlike Tyr288 and Tyr437) substitution with a Phe resulted in a 200-fold decrease in substrate oxidation rate, implying that the likely elevated redox potential of the Tyr374 radical, arising from its H–bonding interaction with the Asp385-H_2_O unit (inset Fig. 1), facilitates an effective electron-transfer pathway with a bulky substrate, thus playing a functional role.^35^

The second A-type DyP homolog present in *S. lividans*, DtpAa, possess 5 Tyr (2 more than in DtpA) and 7 Trp (1 more than DtpA) residues. The X-ray crystal structure of DtpAa reveals no Tyr residues to be in proximity to a proton acceptor, and only Tyr272 is structurally conserved with Tyr288 in DtpA (Fig. 1).^36^ Notably, Tyr374 is replaced in DtpAa by a Phe (Phe347 DtpAa numbering), but Asp385 (Asp358 DtpAa numbering) is conserved, albeit with the structural H_2_O molecule absent.^36^ Stopped-flow absorbance spectroscopy is consistent with compound I formation in both A-type DyPs, followed in the absence of a substrate, by rapid decay to a species possessing wavelength absorbance features consistent with compound II.^34,36^ However, the identification of a radical site(s) in DtpAa has not yet been experimentally determined.

Here we show that the locations of the Tyr residues in DtpAa do not alone determine the location of a stable YO˙ species. By engineering the distal Tyr residue arrangement of DtpA into DtpAa, including the creation of a nearby proton acceptor, does not yield a stable YO˙ species in DtpAa. Only after further protein engineering on the proximal heme side of the protein scaffold did the expected YO˙ species become populated on the distal side of the heme in DtpAa. To account for these observations, we have constructed a kinetic model which includes radical migration pathways both on the proximal and distal sides of the heme. Remarkably, simulations of the model demonstrate that only a small, factor of 10-change in a rate constant, is required to select which migration pathway the porphyrin π-cation radical of compound I takes.

## Experimental

### Site-directed mutagenesis, heterologous expression, and purification of DtpAa

The single, Y345F, double Y345F/F347Y, and triple Y345F/F347Y/Y389F variants of DtpAa were constructed using a site-directed mutagenesis strategy based on the QuikChange method (see ESI† for details). The heterologous expression of wild-type (WT) DtpAa and the three variants was carried out in the *Escherichia coli* strain BL21(DE3) as described previously along with the purification strategy outlined in ESI†.^46^

### Protein preparation

All DtpAa proteins were exchanged into the desired buffer for experiments using desalting or ultracentrifugation methods. Stock protein concentrations were determined from the absorbance at 280 nm measured in a Cary 60 UV-Vis spectrophotometer, using an extinction coefficient (*ε*) at 280 nm of 46 057 M^−1^ cm^−1^ for WT DtpAa and the Y345F/F347Y variant and 44 567 M^−1^ cm^−1^ for the Y345F and the Y345F/F347Y/Y389F variant. Hydrogen peroxide 30% (Sigma-Aldrich) was diluted to a working stock concentration of ∼10 mM, with the concentrations of subsequent dilutions determined spectroscopically using an *ε*_240 nm_ of 43.6 M^−1^ cm^−1^.^47^

### Electron paramagnetic resonance spectroscopy

A Bruker EMX and a Bruker E500 EPR spectrometers (both X-band) were used, each combined with Oxford Instruments liquid helium systems, to measure low temperature continuous wave (CW) EPR spectra. Wilmad SQ EPR tubes (Wilmad Glass, Buena, NJ) with OD = 4.05 ± 0.07 mm and ID = 3.12 ± 0.04 mm (mean ± range) were used. Samples frozen in a set of these tubes yielded very similar intensities of EPR signals - with only ∼1–3% random error. EPR spectra of a blank sample (frozen water) measured at the same set of instrumental conditions were subtracted from the DtpAa spectra to eliminate the background baseline EPR signal. In the experiments which required subtraction of the spectra detected in a ∼10–50 mT field range, corrections for spectra position along the magnetic field were introduced based on the specific microwave frequency recorded for each spectrum. The instrumental set-up used to record spectra were as follows: temperature, *T*_meas_ = 10 or 25 K; microwave (MW) frequency was slightly different for individual samples due to variation of the EPR tube sizes but when averaged (over the samples reported) was *ν*_MW_ = 9.47 ± 0.008 GHz; modulation frequency *ν*_M_ = 100 kHz; modulation amplitude *A*_M_ = 0.5 mT; MW power *P*_MW_ = 3.16 mW; sweep rate *R*_S_ = 2.275 mT s^−1^; time constant *τ* = 81.92 ms; single scan NS = 1. To characterise the EPR signal arising from the YO˙ species, in a smaller magnetic field range, the MW power and the sweep rate were *P*_MW_ = 0.05 mW and *R*_S_ = 0.325 mT s^−1^, respectively.

### Time course sample preparation for EPR spectroscopy measurements

Two methods were used to prepare the time course samples of 40 μM DtpAa in 50 mM sodium acetate, 150 mM NaCl, pH 5.0, reacting with H_2_O_2_. The first involved the addition of a stock H_2_O_2_ solution to give a desired final concentration – either 40 μM the same as the final concentration of DtpAa, or 400 μM (a 10-fold excess), from which an aliquot of the reaction mixture was drawn, placed in an EPR tube and frozen in a cooling bath consisting of methanol kept on dry ice (∼195 K). This method provides the reaction time (the time lapse from components mixed to the EPR tube dropped to the methanol) of ≥11 s. In the second method, samples with reaction times of between 4 and 10 s were prepared by placing the stock H_2_O_2_ solution (at a volume to give a final concentration of 400 μM) inside the tip of a plastic tubing connected to a syringe. The tubing + syringe was then used to draw the DtpAa sample from an EPR tube and subsequently release the mixture back to the EPR tube, followed by immediate freezing in the methanol cooling bath. For all time courses, replicate samples were prepared, as well as multiple repeats using different protein batches.

### EPR spectra deconvolution, simulation and quantitation

The procedure of spectra subtraction with variable coefficient^48^ was used to separate the two high-spin (HS) ferric heme forms (HS_narrow_ and HS_wide_ EPR signals). To relay intensities of these signals to specific concentration, *i.e.* in μM heme, the two signals were simulated by using SimFonia (Bruker) and simulated spectra were double-integrated over the full range of magnetic field values of the signal (from 90 to 460 mT). The sum of the second integrals of the two forms in the control sample was normalised to the protein concentration (40 μM) as determined by UV-vis spectrometry measurements. The same procedure of spectra subtraction with variable coefficient was used to deconvolute the EPR spectra in the free radical range of the magnetic field (∼20 mT around *g* = 2) into the individual EPR signal components, referred to as ‘pure line shapes’, as well as to measure intensities of the components in the EPR time courses. The second integrals of the pure line shapes were referred to the second integral of the EPR spectrum of a concentration standard (200 μM Cu^2+^). The simulation of the free radical EPR signal was performed by the EasySpin package^49^ with the input parameters determined using our algorithm for finding simulation parameters for tyrosyl radical EPR spectra, TRSSA-Y v2.^35,50^ To generate the parameters needed to simulate a Tyr radical EPR line shape, TRSSA uses two inputs, the tyrosyl radical phenoxyl ring rotation angle (*θ*), measurable from X-ray crystal structure coordinates, and the spin density on the Tyr C1 atom (*ρ*_C1_).^50^

### X-ray structure determination and refinement

The ferric DtpAa variants were crystallised using the hanging drop vapour diffusion method (drop size 2.4 μl) with incubation at 18 °C. Protein solutions (15 mg ml^−1^) were mixed with an equal volume (1.2 μl) of precipitant solution consisting of 100 mM HEPES pH 7.5, and 20% PEG 4000 (Sigma-Aldrich). Single rhombohedron crystals (dimensions ∼200 × 150 × 150 μm) grew within 2 days and were used for data collection following cryo-protecting in a precipitant solution containing 20% glycerol (Fisher), and flash-cooled by plunging into liquid nitrogen. X-ray diffraction data was collected at Diamond Light Source (DLS) beamline I04 at 100 K and a wavelength of 0.9795 Å. Diffraction data were integrated, scaled and merged using the xia2 DIALS pipeline. All structures were refined using Refmac5^51^ in the CCP4i2 suite^52^ with ferric DtpAa WT (PDB code 6TB8^36^) used as the input model and with model building between refinement cycles in Coot.^53^ Riding hydrogen atoms were added during refinement. The structures were validated using the Molprobity server,^54^ the JCSG Quality Control Server and tools within Coot.^53^ A summary of data collection and refinement statistics are given in Table 1. As we are looking at the positions of sidechains some distance away from the heme, the iron redox state in the final models is not relevant. However, based on the high flux required to obtain these high-resolution data sets we would expect the absorbed dose to be high enough to reduce the heme to the ferrous state.^55^

**Table:** Crystallographic processing and refinement statistics for *S. lividans* DtpAa variants. Values in parenthesis are for the highest resolution shell

	Y345F	Y345F/F347Y	Y345F/F347Y/Y389F
Space group	*P*2_1_	*P*2_1_	*P*2_1_
Unit cell (Å)	*a* = 72.02, *b* = 67.79, *c* = 73.02, *β* = 105.66°	*a* = 71.04, *b* = 68.13, *c* = 73.05, *β* = 105.42°	*a* = 71.85, *b* = 67.93, *c* = 74.48, *β* = 105.46°
Resolution (Å)	70.31–1.23	70.42–1.27	58.26–1.50
Outer shell (Å)	1.25–1.23	1.29–1.27	1.53–1.50
Number of reflections	191 401 (7298)	174 878 (8482)	110 533 (5452)
*R*_merge_	0.093 (0.94)	0.066 (1.519)	0.078 (1.34)
*R*_pim_	0.070 (0.84)	0.036 (0.93)	0.049 (0.84)
Mn(I/sd)	8.2 (1.6)	9.0 (0.6)	10.9 (1.1)
CC_1/2_	0.996 (0.36)	0.999 (0.34)	0.999 (0.558)
Completeness (%)	97.8 (75.8)	99.0 (83.0)	100 (100)
Redundancy	4.8 (2.8)	5.0 (3.7)	6.9 (6.9)
*R*_work_	0.133	0.152	0.161
*R*_free_	0.159	0.193	0.189
RMSD bond lengths (Å)	0.011	0.014	0.015
RMSD bond angles (°)	1.748	1.842	2.005
Ramachandran most favoured (%)	97	96	97
PDB accession code	8OLH	8OLP	8OMC

### Calculating hole hopping pathways

The python-based programme EHPath was used to identify hole hopping routes and calculate the timescale for hole hopping from a donor to an acceptor.^56^ X-ray crystal structures of DtpA (PDB code 6GZW),^35^ DtpAa (PDB code 6I43)^57^ and the DtpAa variants determined in this work were used as inputs, with donor, bridge and acceptor files prepared for each X-ray structure as described in reference.^56^ The mean residence time for the charge on the hole hopping route (*τ*_M_ s) was calculated using eqn (1)–(3) and (5), given in reference.^56^

### Kinetic model construction and simulation

Construction of the kinetic model was informed by experimental data from stopped-flow kinetics, hole hopping calculations, and the EPR time courses, along with literature values for TrpNH˙^+^ p*K*_a_.^44,45^ Simulation of the model was performed using ProK (Applied Photophysics, UK).

## Results & discussion

### Transient populations of EPR signals are observed on reacting ferric DtpAa with H_2_O_2_

**Fig. 2:**
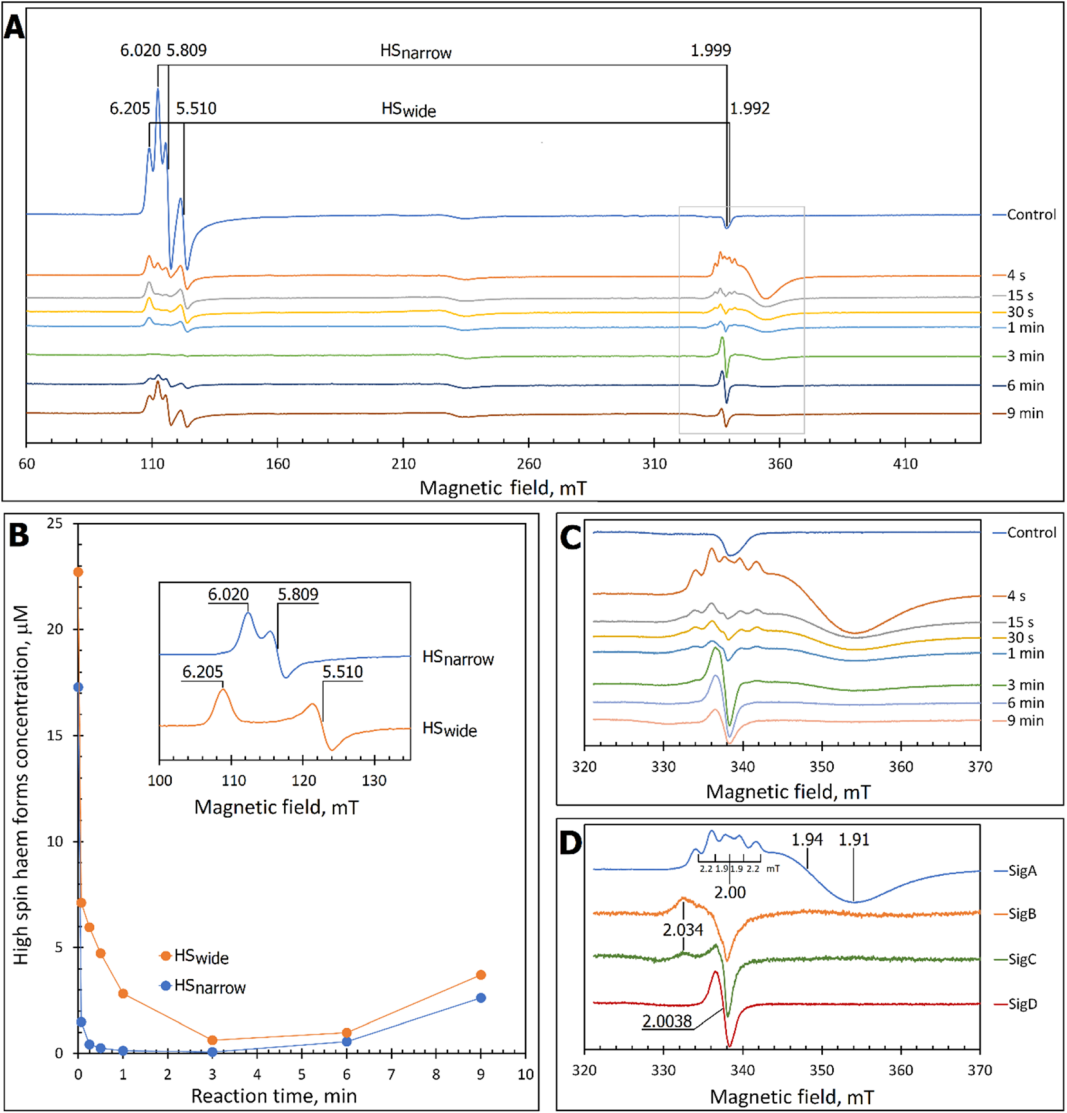
X-band CW EPR spectroscopy of DtpAa upon reaction with H_2_O_2_ at 10 K and pH 5.0. (A) The EPR spectra of DtpAa before (Control) and at different times after mixing with 400 μM H_2_O_2_. The *g*-values of the two HS ferric heme forms, HS_narrow_ and HS_wide_, are indicated and are consistent with those previously reported.^36^ The signal at ∼230 millitesla is a background contribution. (B) The kinetic dependences averaged over two independent experiments of the two HS ferric heme forms. The inset gives the respective line shapes of the two HS signals, in the *g* = 6 region, extracted by spectral subtraction with variable coefficient,^48^ formulated as follows: HS_narrow_ = (Control) − 3.3 × (4 s); HS_wide_ = (4) − 0.084 × (Control). (C) Detailed view of the spectral region covering the field interval of 320–370 mT within the grey frame in (A). All instrumental conditions used were the same as in (A) (see Experimental section) but with a slower *R*_S_ = 0.325 mT s^−1^. (D) The spectral components (pure line shapes) contributing to the EPR spectra shown in C. All signals (SigA to SigD) were obtained by using the procedure of spectra subtraction with variable coefficient,^48^ formulated as follows; SigA = (4 s) − 0.23 × (Control); SigB = (15 s) − 0.61 × (4 s) − 0.145 × (1 min); SigC = (1 min) − 0.61 × (4 s) − 0.392 × (15 s); SigD = (6 min) − 0.337 × (3 min). The *g*-values of spectral points of interest are indicated and the distances between the hyperfine components of SigA, are indicated in millitesla.

At pH 5 the EPR spectrum of ferric DtpAa reveals two high-spin (HS) heme species with distinct *g*-factors and zero field splitting rhombicity parameters (E/D), which we have previously reported and assigned as HS_narrow_ and HS_wide_ (Fig. 2A and B).^36^ Upon addition of 10-fold excess H_2_O_2_, and in the absence of a substrate, the HS signals display distinct kinetic profiles (HS_narrow_ decaying faster than HS_wide_, Fig. 2B), consistent with solution state stopped-flow kinetics.^36^ As these HS signals decay, new signals in the free radical *g* = 2 region of the EPR spectrum appear (Fig. 2A and C). By applying a spectral subtraction with variable coefficient approach,^48^ the pure EPR signals populating the *g* = 2 region over the reaction time course were extracted (Fig. 2D), revealing four distinct transient paramagnetic species, which we have named SigA, SigB, SigC and SigD.

### SigA and SigB are compound I-like species

SigA is wider than typical EPR signals of protein-based free radicals, it is centred at *g* = 1.94 (Fig. 2D) and does not saturate with increasing microwave power (Fig. S1†), as would be expected if the radical was polypeptide based. Instead SigA is affected by a factor which significantly increases the relaxation of the paramagnetic centre, for example a metal ion, as would be the case for a compound I species, in which the π-cation radical of the porphyrin is in a strong electron exchange interaction with the *S* = 1 of the Fe(iv)=O heme. However, the line shape and particularly the presence of hyperfine structure in the signal (Fig. 2D), do not conform with any reported EPR spectrum of compound I in a range of heme enzymes,^58–67^ including also the compound I EPR signal reported for DtpA.^35^ Variation amongst EPR compound I line shapes is not unusual and can be attributed to the changeable contribution of the exchange parameter *J* to the spin Hamiltonian that describes the interaction between the Fe(iv)=O electron spin *S* = 1 and Por˙^+^ spin *S*′ = 1/2. However, what is unusual, is the presence of the hyperfine features – the lines separated by 1.9–2.2 mT intervals around *g* = 2 (Fig. 2D), which to our knowledge are unprecedented in a compound I EPR signal. Further investigation into these hyperfine signals is merited and is underway using Q- and W-band EPR methods.

SigB has an asymmetric EPR line shape, which is very similar to the 9 GHz EPR spectrum reported for compound I in yeast CcP described as a (Fe(iv)=O[Trp˙^+^]).^68^ The CcP compound I is historically termed compound ES, to highlight that the cation free radical is located not on the heme π-system but on a protein residue (Trp191 on the proximal side of the heme in CcP) which is still in an exchange interaction with the *S* = 1 Fe(iv)=O^69,70^ resulting in the distinct EPR line shape.^71^ Therefore, based on the EPR line shape similarity of SigB with compound ES of CcP, we assign SigB to a compound ES-like species, which we name compound I*.

### SigC and SigD display EPR line shapes consistent with uncoupled protein radicals

A classical EPR signature of an amino acid free radical, non-exchange coupled to a Fe(iv)=O heme would be centred close to *g* = 2, not wider than ∼5 mT and subjectable to microwave power saturation. Both SigC and SigD exhibit these characteristics.

SigC has a typical line shape of an axially symmetrical free radical species with a local maximum at *g* = 2.034 (the parallel component) and the main feature close to *g* = 2.005 (two perpendicular components, Fig. 2D). Such line shape has been reported for organic molecules as well as proteins and is assigned to a peroxyl radical (ROO˙).^72–76^ These radicals are often found on Trp residues,^76–78^ resulting from reaction of the Trp˙ with O_2_ to form a Trp-OO˙ adduct.

SigD is a symmetrical 1.8 mT wide singlet, without any visible hyperfine features or *g*-value anisotropy. Such an EPR signal has been observed at late points of the time courses of several heme proteins and enzymes reacting with H_2_O_2_. No matter which enzyme it is, the EPR signal is the same plain singlet caused by a superimposition of signals from many different protein-bound radicals formed by intra- and/or intermolecular radical transfer from the primary site to a range of different sites, and is essentially a marker of oxidative damage.^79^

### The kinetics of the cryo-trapped EPR species are consistent with those of the solution state

**Fig. 3:**
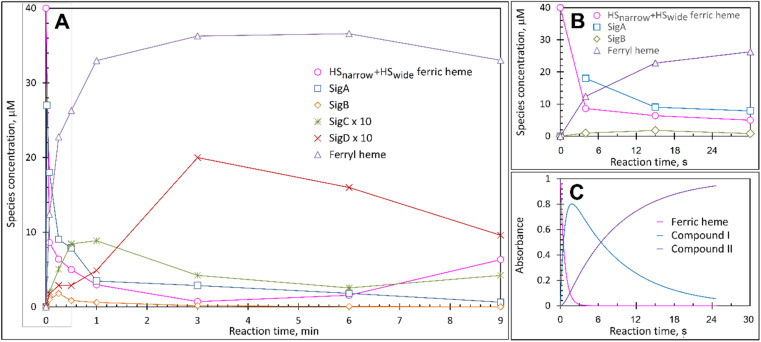
The kinetics of the active species in the reaction of DtpAa with H_2_O_2_ as observed by and EPR and UV-vis spectroscopies. (A) Time course of the paramagnetic species identified by EPR spectroscopy after 40 μM DtpAa was mixed with 400 μM H_2_O_2_, at pH 5 (both concentrations are final in the mixture). The total HS ferric heme forms (the sum of HS_narrow_ and HS_wide_) are plotted and SigC and SigD have been multiplied by a factor of 10. The curve representing the ferryl state of the heme, which is EPR invisible, was calculated as 40 μM – all EPR detectable heme forms. (B) The kinetic data in the grey rectangle in (A) plotted to enable a convenient comparison with the UV-vis spectroscopy data (C) obtained using stopped-flow kinetics in solution at 25 °C.

Each species detected in the EPR spectrum (Fig. 2) can be expressed in concentration (μM), which when plotted against time, provides a kinetic overview of the paramagnetic species population (Fig. 3A). As the HS ferric heme species diminish, SigA (compound I) is forming fast, with its highest concentration (∼26 μM) found at the shortest time point collected (4 s), indicating that the concentration of the species responsible for SigA might be higher at the reaction time shorter than 4 s (Fig. 3A). In contrast, SigB (compound I*), shows an increase over the first two data points reaching a maximum concentration of 1.2 μM (an order of magnitude lower than SigA) by 15 s before starting to decrease (Fig. 3A). Concomitantly with SigB, SigC (peroxyl radical ROO˙, possibly Trp-OO˙) appears (Fig. 3A). It reaches a maximum concentration of 0.9 μM at 60 s over the period SigB decays and on the same order of magnitude as SigB (Fig. 3A). Therefore, it is possible that the SigB species has reacted with O_2_ and is transformed to a Trp-OO˙ adduct yielding SigC. Finally, SigD, fully in agreement with its assignment as “multi-site” protein radical species is the last to appear and reach a maximum concentration of ∼2 μM at 3 min (Fig. 3A). This maximum is achieved over the period of SigC decrease.

A comparison of the early time course EPR kinetic data of SigA and SigB (Fig. 3B), with that of absorbance spectroscopy obtained from stopped-flow kinetics (Fig. 3C) reveals good agreement between solution and frozen states. The ferric heme (HS_narrow_ and HS_wide_) signals decrease on H_2_O_2_ addition, SigA (compound I) is forming and decaying over the fast period of the HS ferric heme decay, with SigB becoming populated, albeit to a low level as SigA decreases, with the decay of SigA and SigB linked to Fe(iv)=O (compound II) formation (Fig. 3B and C).

### Calculations of hole hopping pathways in DtpA and DtpAa

**Fig. 4:**
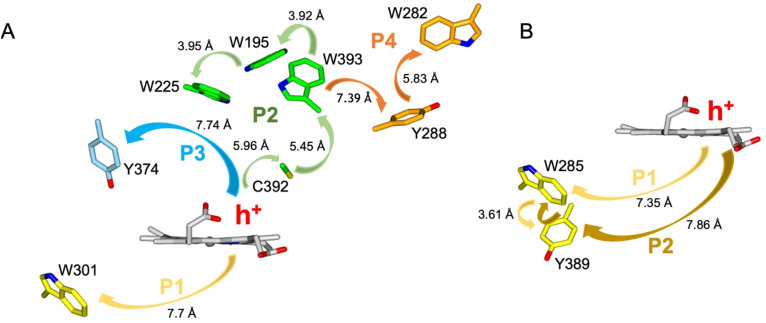
Hole hopping routes in DtpA (A) and DtpAa (B). EHPath ^56^ was used to calculate hole hopping pathways (P) starting from the hole (h^+^) formed on the heme upon reaction with H_2_O_2_. The arrows represent the direction of h^+^ transfer within each pathway. The minimum edge-to-edge distance (Å) between donor and acceptor (*R*_ee_) is indicated for each pathway.

The EPR signals populated on the DtpAa time course following reaction with H_2_O_2_, are distinctly different from those previously reported for DtpA,^35^ where no SigB or SigC signals were detected.^35^ Instead as compound I decays, an EPR signal attributed to a stable YO˙ species appears in DtpA.^35^ No such signal was detected in DtpAa. Therefore, as the YO˙ species in DtpA is assigned to Tyr374, which is absent in DtpAa, we expect that by creating the DtpA distal Tyr arrangement in DtpAa, a stable YO˙ species would form. Prior to creating the site-directed variants in DtpAa, hole hopping pathways in DtpA and DtpAa were explored using the EHPath program.^56^ The X-ray crystal structures of DtpAa and DtpA were used as inputs, with the heme defined as the donor, from which an electron or hole will move. EHPath computes the dominant charge hopping pathway using all electron-transfer active (ETA) residues (Tyr, Trp, Cys and Met) based on mean residence times (*τ*_M_) of the transferring charge along the hopping pathways.^56^ For DtpA and DtpAa, the fastest hole hopping pathways and *τ*_M_ values are reported in Table 2, and illustrated structurally in Fig. 4A and B. Notably for both enzymes, the fastest pathways, (*τ*_M_ 10^−3^ s) end at Trp residues, with Trp301 and Trp285 in DtpA and DtpAa, respectively, located on the proximal side of the heme (Fig. 4). Two slower pathways (*τ*_M_ 10^−2^ s), P3 and P4, are also calculated in DtpA, with each of these routes ending at a Tyr residue on the distal side of the heme (Fig. 4A, Table 2). EPR studies with DtpA,^35^ revealed no experimental evidence for a radical Trp species, with the experimentally observed YO˙ species in DtpA consistent with the P3 calculation ending at Tyr374 (Table 2).^35^ In DtpAa the proximal Trp285 is in close proximity to Tyr389, with the aromatic rings in this redox active dyad adopting a T-shaped orientation (Fig. 4B). The calculated P1 and P2 pathways in DtpAa cycle around this redox active dyad (Table 2), whereas in DtpA, Tyr389 is substituted for a Phe. The substitution to a non-ETA residue will have consequences for stabilising a radical site on Trp301 and could be a reason for the observation of a SigB signal in DtpAa but not in DtpA.

Mean residence time (*τ*_M_) of the hole for the fastest hole hopping pathways (P) in *S. lividans* DtpA, DtpAa and DtpAa variants

**Table:** 

DtpA	*τ*_M_ s	DtpAa	*τ*_M_ s
P1: HEM-W301	1.4 × 10^−3^	P1: HEM-W285	1.3 × 10^−3^
P2: HEM-C392-W393	2.2 × 10^−3^	P1: HEM-W285-Y389	1.3 × 10^−3^
P2: HEM-C392-W393-W195	2.4 × 10^−3^	P2: HEM-Y389	6.8 × 10^−3^
P2: HEM-C392-W393-W195-W225	2.4 × 10^−3^	P2: HEM-Y389-W285	6.8 × 10^−3^
P3: HEM-Y374	1.7 × 10^−2^		
P4: HEM-C392-W393-Y288	5.0 × 10^−2^		
P4: HEM-C392-W393-Y288-W282	5.3 × 10^−2^

**Table:** 

DtpAa Y345F	*τ*_M_ s	DtpAa Y345F/F347Y	*τ*_M_ s
P1: HEM-W285	1.2 × 10^−3^	P1: HEM-W285	1.3 × 10^−3^
P1: HEM-W285-Y389	1.2 × 10^−3^	P1: HEM-W285-Y389	1.3 × 10^−3^
P2: HEM-Y389	6.5 × 10^−3^	P2: HEM-Y389	6.5 × 10^−3^
P2: HEM-Y389-W285	6.5 × 10^−3^	P2: HEM-Y389-W285	6.5 × 10^−3^
		P3: HEM-Y347	2.3 × 10^−2^

**Table:** 

DtpAa Y345F/F347Y/Y389F	*τ*_M_ s
P1: HEM-W285	1.3 × 10^−3^
P2: HEM-Y347	2.9 × 10^−2^

### Engineering a pre-selected destination for a Tyr radical

**Fig. 5:**
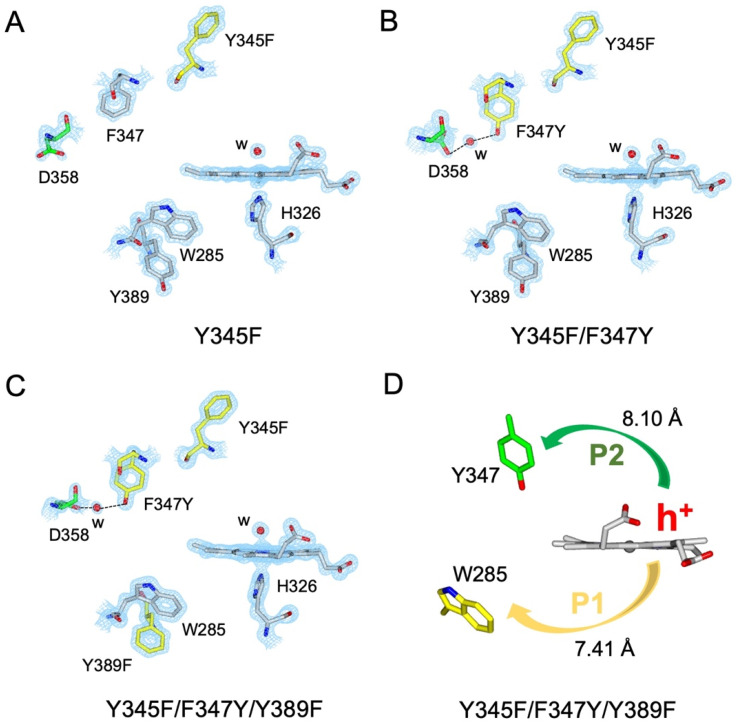
X-ray crystal structures of DtpAa variants. (A–C) 2*F*_o_–*F*_c_ density maps contoured at 1.0 *σ* for the heme and selected residues. Aromatic residues substituted in each variant are coloured in yellow, water molecules (w) are indicated by a red sphere, and the H-bond network formed in the Y345F/F347Y and Y345F/F347Y/Y389F variants is indicated by dashed lines. (D) Calculated hole hopping pathways P1 and P2 for the triple variant starting from the hole (h^+^) that transfers from the heme on forming compound I. The minimum edge-to-edge distance (Å) between donor and acceptor (*R*_ee_) is indicated for each pathway.

A suite of DtpAa variants was constructed to test whether a predicted stable YO˙ species could be formed. The Y345F variant, creates a DtpAa protein with no Tyr residues within 15 Å of the heme on the distal side with the double variant, Y345F/F347Y, mimicking the distal heme Tyr arrangement as in DtpA. High-resolution X-ray crystal structures (Fig. 5, Table 1) determined to 1.23 Å (Y345F) and 1.27 Å (Y345F/F347) resolutions, corroborated the desired amino acid changes. The X-ray crystal structure of the double variant reveals that the Asp358 side chain undergoes a positional change, relative to that observed in the Y345F variant and WT DtpAa, enabling for a H-bond network to form with a newly appeared H_2_O molecule and the Tyr347 phenol group (Fig. 5). This arrangement mimics the proton acceptor architecture identified in DtpA (inset Fig. 1), considered important for creating a stable YO˙ species.^35^ The triple variant, Y345F/F347Y/Y389F, now mimics the Tyr arrangement on the distal heme side and the proximal Trp environment of DtpA. The X-ray crystal structure of the triple variant was determined to 1.50 Å resolution (Fig. 5 and Table 1), confirming the presence of the introduced proximal Phe, and further corroborated the integrity of the structural H_2_O in proximity of Tyr347 and Asp358 (Fig. 5).

Using the DtpAa variant X-ray structures as inputs, hole hopping pathways were calculated using EHPath (Table 2). Not surprisingly, the fastest hole hopping pathways (*τ*_M_ 10^−3^ s) in the Y345F and double variant remain dominated by the proximal Trp/Tyr dyad (Table 2). However, in the double variant, a hole hopping pathway is calculated (P3, Table 2), with a *τ*_M_ value on the same order of magnitude to that calculated for the Tyr374 pathway in DtpA (Fig. 4A and Table 2). Thus, the introduction in DtpAa of a Tyr at position 347 distal to the heme facilitates a new hole hopping pathway. Finally, disrupting the charge distribution of the Trp285/Tyr389 dyad in the triple variant, results in only two calculated routes, one to the proximal Trp and the second to the engineered distal Tyr347 (Fig. 4A and Table 2).

The electronic absorbance spectra for the ferric Y345F, double and triple variants are reported in Fig. S2,† together with the spectral changes that occur upon stoichiometric addition of H_2_O_2_. It is evident from these data, that for the double and triple variant the life-time of compound I increases. This was further corroborated from stopped-flow experiments, where on mixing with excess H_2_O_2_, the kinetics of compound I formation displayed a similar pattern of rate constant (k_obs1_) dependence on the H_2_O_2_ concentration to that observed for the WT DtpAa,^36^ but the H_2_O_2_ concentration independent decay of compound I to II (k_obs2_) decreased on the order of Y345F (0.19 s^−1^) > Y345F/F347Y (0.10 s^−1^) > Y345F/F347Y/Y389F (0.07 s^−1^). Taken together these data support the conclusion that the compound I lifetime/population is increased in the double and triple variants.

### Disruption of the proximal Trp/Tyr dyad is required to form a stable YO˙ at the engineered Tyr347 position in DtpAa

**Fig. 6:**
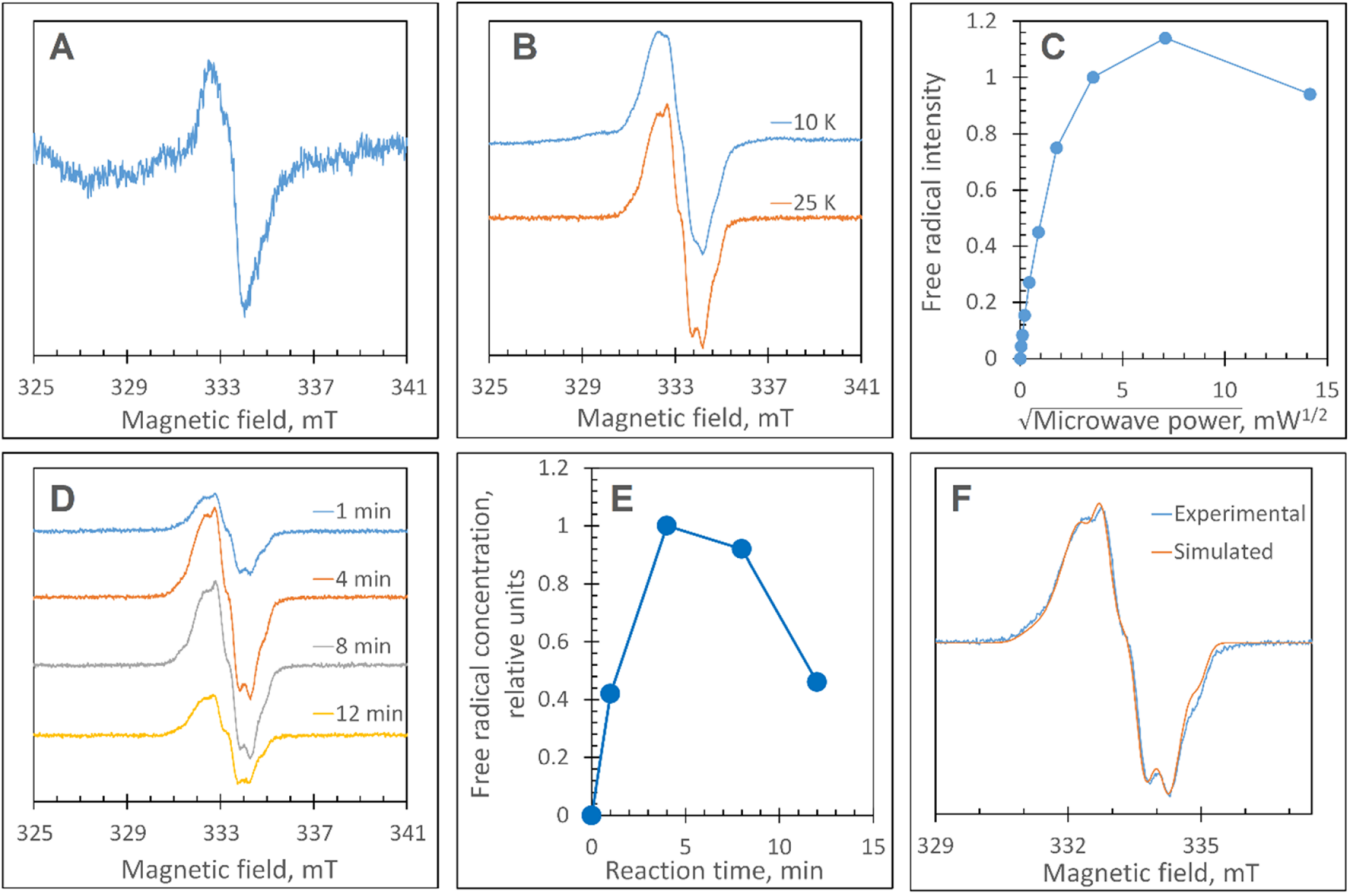
EPR characterisation of the YO˙ species. (A) The free radical EPR signal detected in the DtpAa double variant, Y345F/F347Y, treated with H_2_O_2_. The signal is a result of spectra subtraction with variable coefficient and was obtained as follows: 0.26 × (180 s) + 0.68 × (60 s) − 0.47 × (5 s). In brackets in the formula, are the EPR spectra measured of the samples of 40 μM Y345F/F347Y DtpAa mixed with 400 μM H_2_O_2_ (both concentrations are final – in the mixture) frozen at the times after mixing indicated. (B) The free radical EPR signal formed on the DtpAa triple variant, Y345F/F347Y/Y389F, frozen 6 min after addition of 10-fold excess H_2_O_2_ shows a better line resolution when measured at 25 K as compared to 10 K. (C) The microwave power saturation dependence of the free radical EPR signal (as detected in B). The signal intensities values have been found with the use of the subtraction with variable coefficient. A potentially more accurate procedure of measuring the second integrals of the signals was not easy to implement, as the free radical signal is located on a different EPR signal. (D) The kinetic set of the free radical EPR signals of the 40 μM triple variant DtpAa mixed with 40 μM H_2_O_2_ and frozen various times thereafter (indicated). The spectra have been measured at 25 K whereas the other instrumental conditions were as in A; the spectra have been aligned along the magnetic field to common g values – on the basis of the microwave frequencies used to record each spectrum. (E) The free radical kinetics in relative units as measured from the EPR spectra shown in (D), when using the spectra subtraction with variable coefficient. (F) A TRSSA-Y supported simulation of the free radical EPR signal shown in B (25 K). The TRSSA input parameters were *ρ*_C1_ = 0.36 and *θ* = 56.5° (or the complimentary angle of 61.5°). The full set of the Hamiltonian parameters generated by TRSSA-Y for this input is reported in Table S1†.

The Y345F variant showed no differences to WT DtpAa, in the *g* = 2 region of the EPR spectrum (Fig. S3 and S4†). In the case of the double variant (mimics the Tyr arrangement of the distal heme in DtpA), a lowly populated EPR signal, with a maximal intensity at ∼3 min was detected (Fig. 6A). The line shape and position, albeit of a low signal-to-noise ratio, are consistent with a possibility of the signal being caused by a YO˙ species. By contrast to this low intensity free radical signal in the double variant, the triple variant (mimics DtpA on both distal and proximal heme) gave a strong EPR signal in the *g* = 2 region (compared to the double variant) with a line shape (Fig. 6B) and microwave power saturation behaviour (Fig. 6C) consistent with being caused by an uncoupled (not coupled to heme iron) protein radical.^76^ The power saturation dependence strongly implies a non-homogenously broadened linewidth, and the measurements at 25 K, rather than at 10 K, yielded a better resolution of the hyperfine features (Fig. 6B). The 25 K spectrum is very similar to the DtpA spectrum we reported previously.^35^ Under single turn-over conditions, the radical species is fully formed within the first 1 min of the time course and thereafter decays (Fig. 6D and E). Spectral simulation of the radical signal was successfully achieved using TRSSA-Y.v2,^35^ unambiguously confirming it to be caused by a YO˙ species (Fig. 6F). Comparison of the *θ* value found from the simulation, with the *θ* angles determined from the Tyr residues in the X-ray structure of the triple variant are reported in Table 3. All 4 Tyr residues have *θ* angles within 5° of each other (Table 3). As we have reported previously for DtpA,^35^ structural homogeneity amongst Tyr side chain rotational conformations makes an assignment of the YO˙ species to a specific Tyr ambiguous. For DtpA mutational analysis was required to demonstrate that Tyr374 was the YO˙ species location.^35^ Therefore, based on the near identical line shapes of the YO˙ species in the triple variant of DtpAa, and WT DtpA,^35^ we assign the YO˙ species in the triple variant to the engineered Tyr347 – the position homologous to 374 in DtpA.

**Table:** The tyrosine ring rotation angle (*θ*) for the Tyr residues in the DtpAa triple variant, calculated as explained in ref. 50 form the dihedral angles (*ϕ*_2_ = *C*_α_ − *C*_β_ − C1 − C2 and *ϕ*_6_ = *C*_α_ − *C*_β_ − C1 − C6) determined from the X-ray structure. The last two columns report the difference between *θ* and the two complementary angles^a^ found when using TRSSA-Y

Tyr number	*ϕ*_2_ (^o^)	*ϕ*_6_ (^o^)	*θ* (^o^)	|*θ* − 56.5°|	|*θ* − 61.5°|
116	−88.49	90.44	59.025	2.525	2.475
272	−87.82	85.23	61.295	4.795	0.205
347	−96.14	87.37	64.385	7.885	2.885
366	−89.5	88.31	60.595	4.095	0.905

a The *θ* = 61.5° gives an identical simulated spectrum as the *θ* = 56.6° does, due to the symmetry of the two configurations as 56.5° + 61.5° = 118° – the angle between the two methylene protons as shown by DFT calculations.^84^

**Fig. 7:**
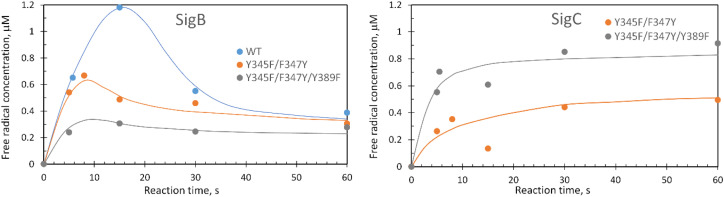
Comparison of SigB and SigC formation for the double and triple DtpAa variants. Plots represent the first 60 s of the EPR time course following a 10-fold addition of H_2_O_2_ to a 40 μM DtpAa sample. The solid lines through the data points are to guide the eye.

These data surprisingly demonstrate that when the ETA proximal Trp/Tyr dyad is intact the YO˙ species is populated at a very low level, and it is only by removing the Tyr in the dyad (a Phe in DtpA), that the YO˙ species is formed at a high concentration and at the desired location, Tyr347. This implies that radical migration can be initiated from the porphyrin of compound I *via* two pathways (proximal or distal). A further observation from the EPR time course data, consistent with two pathways, is that the relative population of SigB decreases on the order WT > Y345F/F347Y > Y345F/F347Y/Y389F (Fig. 7A). However, for SigC, an inverse relationship in population compared to SigB is observed (Fig. 7B). An explanation for this inversion in concentrations is further discussed below, following the introduction of a kinetic model and its subsequent simulation.

### Constructing a kinetic model to account for the proximal and distal radical migration pathways

**Fig. 8:**
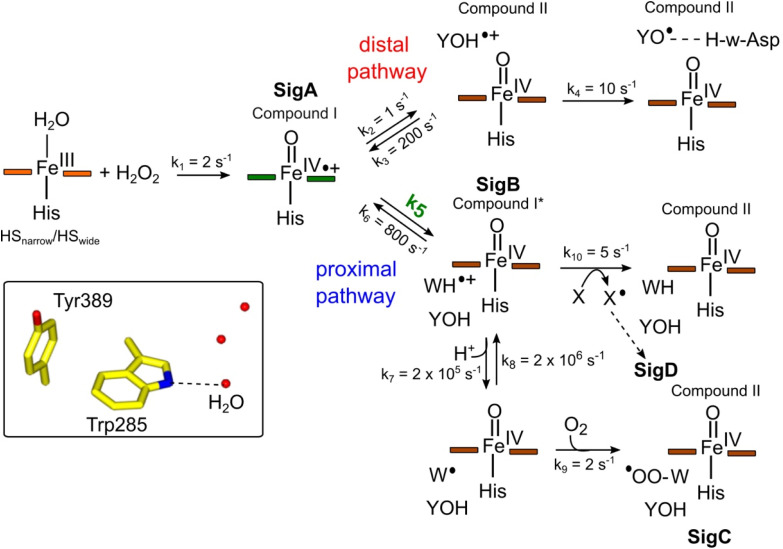
Kinetic model for radical migration in A-type DyPs. The rate constants for most of the individual steps are described in the main text, with the exception of *k*_4_, *k*_9_ and *k*_10_. Values for these rate constants were chosen to give satisfactory time courses that simulate those observed by optical and EPR spectroscopies for the double and triple variants and remain constant across all simulations. The dashed arrow leading to SigD indicates multiple steps as discussed in.^79^ We have used the term compound II for all Fe(iv)=O heme for which the *S* = 1 is not coupled to a radical species. Boxed inset depicts the indole NH–H_2_O H-bond (dashed line) of Trp285 in the proximal Trp/Tyr dyad.

To make a consistent whole of our DtpAa data, bringing together the time courses from optical and EPR spectroscopies, the concentrations and time dependencies of intermediate species, and hole hopping calculations, we have constructed the kinetic model presented in Fig. 8. The model replicates the essential features of our experimental data and provides a simple explanation to account for whether a proximal or distal pathway dominates and controls radical migration from compound I (Fig. 8).

The initial step of the reaction with H_2_O_2_ is with the ferric enzyme to produce compound I, with *k*_1_ (Fig. 8) determined experimentally^36^ and unaffected in the variants. In our model, SigA (compound I) may decay *via* two pathways, both of which involve the compound I porphyrin π-cation radical being quenched by an electron from a nearby ETA aromatic residue. The proximal heme pathway represents DtpAa proteins in which the ETA Trp285/Tyr389 dyad on the proximal side of the heme (*i.e.* WT and the Y345F/F347Y variant) is intact, and electron-transfer from this dyad generates a radical species, which based on the near identical EPR line shape of SigB to that of CcP compound ES,^68^ is consistent with the presence of a coupled Fe(iv)=O Trp285˙^+^ species (compound I*). Deprotonation of the Trp285NH˙^+^ generates a neutral Trp˙ species, which on interaction with O_2_ produces SigC, the peroxyl radical (Fig. 2 and 8). In heme systems exhibiting a Trp coupled compound I*, it has been reported that at later H_2_O_2_ reaction times, the EPR signal evolves to another EPR line shape assigned as Trp-OO˙ species.^80–82^ Thus appearance of SigC is wholly consistent with Trp285˙^+^ reacting with O_2_ and causing disruption of the exchange coupling between the Trp˙^+^ and the Fe(iv)=O heme (Fig. 8). Alternatively, the Trp285NH˙^+^ may be quenched by another amino acid (X in Fig. 8), through either inter- or intramolecular electron-transfer,^79^ which in a single turnover (as simulated in our model below) does not give a detectable signal, but on multiple turnovers, as in the EPR time course, generates SigD (Fig. 8). The distal heme pathway represents DtpAa proteins whereby the electron donated to compound I is from a distal YOH (*i.e.* Tyr347) to generate YOH˙^+^, which deprotonates to form a YO˙ species observable by EPR spectroscopy. This pathway is present in the double and triple variants as well as in DtpA.^35^

As the calculated *τ*_M_ values are functions of the sum of the two rate constants (forward and reverse),^56^ we have selected in the model values for *k*_2_, *k*_3_, *k*_5_, and *k*_6_, which are consistent with the *τ*_M_ values reported in Table 2, and also allow for realistic simulation of our data set (Fig. 8). In both the proximal and distal pathways, we propose that it is the back rate constants (*k*_3_ and *k*_6_) from the aromatic radical cations to the heme that dominate the calculated *τ*_M_ values (Fig. 8). In other words, the radical cation is preferentially located on the heme rather than the ETA aromatic residues, were it otherwise we would observe experimentally (in both the optical and EPR data) far less of compound I. The values of *k*_7_ and *k*_8_ were selected arbitrarily to be very fast so that SigB and the deprotonated Trp˙ species are always at equilibrium, with *k*_8_ being 10-fold faster than *k*_7_ implying that the p*K*_a_ of the dyad is 1 unit above the ambient pH (*i.e.* p*K*_a._ ∼6). This is a reasonable assumption as the indole NH is H-bonded to a H_2_O molecule in DtpAa (*inset*Fig. 8) thus making deprotonation disfavoured, therefore, favouring the equilibrium towards SigB.

The critical test of the model is to assess whether it accounts for our experimentally observed differences between the double and triple variants. In the double variant, although the engineered distal Tyr347 is present, and indeed identified as the YO˙ species in the triple variant, it is minimally populated (Fig. 6A). SigB on the other hand is populated in the double variant (Fig. 7A), indicating that the proximal pathway dominates. However, in the triple variant in which charge distribution over the ETA components of the dyad (Trp285/Tyr389) is disrupted by replacing the Tyr with a Phe, SigB is lowly populated and the YO˙ species (Tyr347) is almost fully populated (Fig. 6). We therefore propose that the switch between these two radical pathways lies within the value of *k*_5_ (Fig. 8), with a larger *k*_5_ value when the proximal ETA Trp285/Tyr389 dyad is present.

### Simulation of the model

**Fig. 9:**
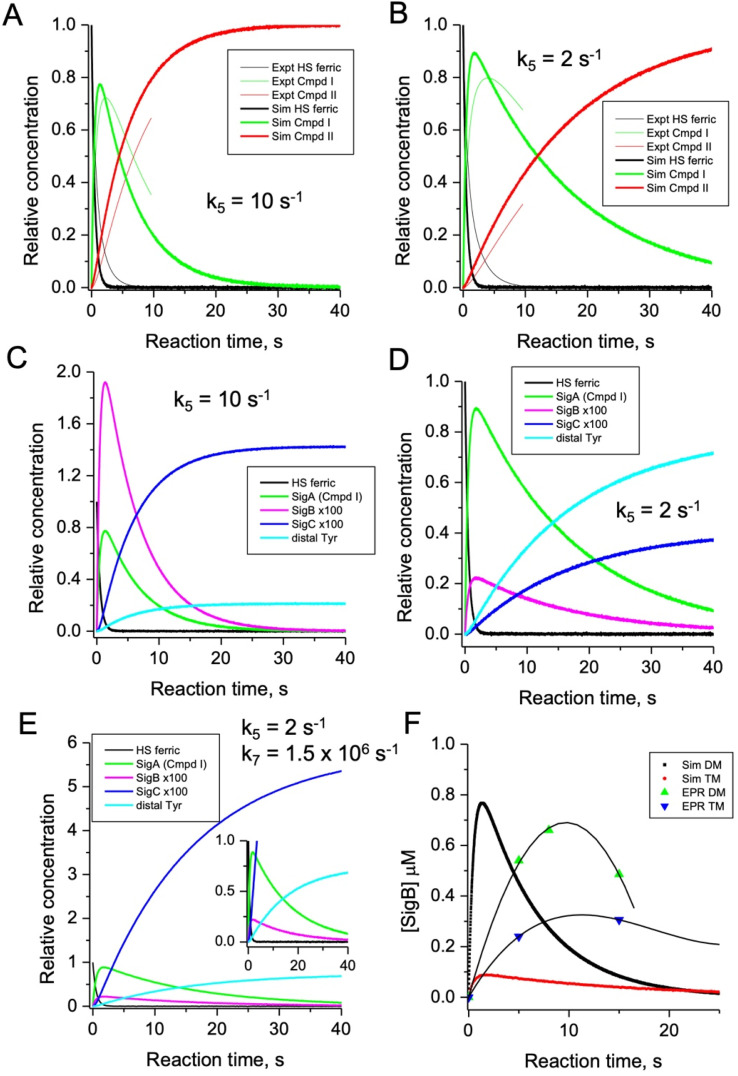
Time course simulations for the proximal and distal pathways of the kinetic model depicted in Fig. 8. The relative concentrations (1.0 for resting state ferric heme) of the observable species in the optical (A and B) and EPR (C and D) spectroscopies have been simulated using a *k*_5_ value as indicated in the respective panel. For the optical simulations (panels A and B) the experimentally obtained stopped-flow data for the double (A) and triple (B) variant, respectively, are overlayed for comparison. Panel (E) is the simulation of the EPR species for the triple variant (distal pathway) by changing *k*_7_ as discussed in the main text. Inset shows the zoomed-in region of the time course simulation. Panel (F) represents an overlay of the simulated and EPR experimental time course data for SigB over the first 20 s for the double (DM) and triple variants (TM), plotted as a function of SigB concentration (μM). The solid lines through the experimental data points are to guide the eye. All simulations are for a single turnover, and therefore no recycling of the heme with excess H_2_O_2_, and as such the simulations only cover the first 40 s and not the several minutes that are followed in EPR.

To test the hypothesis that *k*_5_ is the kinetic switch that pivots the distal or proximal pathways we simulated the model using *k*_5_ values of 20 and 2 s^−1^ for the double and triple variant, respectively, (with all other rate constants as given in the model, Fig. 8). The simulations reveal an excellent correlation with the experimental optical data (Fig. 9A and B). Moreover, both *k*_5_ simulations satisfactorily predict that the decay rate constant of compound I decreases from 0.17 to 0.06 s^−1^ for the double and triple variant, respectively, consistent with experimental evidence of a longer-lived compound I in the triple variant. By varying *k*_5_ as done for the optical data, we also simulated the EPR data (Fig. 9C and D). When *k*_5_ = 2 s^−1^ the distal pathway now efficiently competes with the proximal pathway (*k*_5_ = 20 s^−1^; Fig. 9C) as evidenced by the production of a high population of the YO˙ species and depletion of SigB (Fig. 9D), which is consistent with the experimental EPR observations (Fig. 6 and 7). Thus, decreasing *k*_5_ by a factor of 10 in the triple variant, whilst keeping *k*_6_ constant, means that the equilibrium constant between compound I and compound I* changes by a factor of 10 in favour of compound I. An alteration of this magnitude in equilibrium constant implies that the Trp˙^+^ is destabilised by some 5.7 kJ mol^−1^ on replacing the ETA Tyr of the Trp285/Ty389 dyad with a Phe. Whilst a *k*_5_ value of 2 s^−1^ satisfactorily simulates SigB and the YO˙ species in the triple variant (Fig. 9D), the population of SigC is higher than that simulated in the double variant (Fig. 9C and D) and therefore contrary to the experimental observations (Fig. 7B). SigC as discussed, arises from deprotonating Trp285NH˙^+^ and its subsequent reaction with O_2_ (Fig. 8). In the triple variant Trp285 is no longer interacting with the ETA Tyr which will better delocalise a positive charge. We therefore propose that on replacing the Tyr with a Phe, the charge distribution is now more favourable for deprotonation and thus lower the p*K*_a_. We have affected this in the model by bringing the values of *k*_7_ and *k*_8_ closer, *i.e.* by increasing the value of *k*_7_ to 1.5 × 10^6^ s^−1^. From the resulting simulation the population of SigC now exceeds that of the double variant (Fig. 9E), and now in line with the experimental observation (Fig. 7B). Importantly, the change in p*K*_a_, does not affect the distribution of species between the proximal and distal pathways and thus the optical simulations and that of YO˙ species are unchanged (Fig. S5†). The simulations and the experimental data for SigB are compared in Fig. 9F, where the major features such as the concentrations and the time scales are within the same order of magnitude. Given that the EPR data is captured following freezing we might expect the details of the simulation between the EPR and optical experiments to differ. Finally, although the simulations are semi-quantitative, and do not fit exactly the experimental data, they nevertheless provide a qualitative match and act as aids to understand the mechanism of radical formation and transfer.

## Conclusions

Although the true functional role of DyPs is yet to be elucidated, there is a growing body of experimental evidence to suggest that the oxidative chemistries they are likely to engage in are dictated by their ability to form stable functional radical sites, to oxidise organic substrates. In line with this view, it is noted that across the bacterial and fungal families, DyPs enjoy a higher proportion of Tyr and Trp residues, compared with other peroxidase families, including LiP and VP members.^10^ Furthermore, of the 3 DyP sub-families (A, B, C/D) only members from the A and C/D sub-families have so far been reported to form functionally important Tyr/Trp radical sites following initial reaction with H_2_O_2_. It is notable that no B-type DyP members have been reported to generate stable radical sites *in vitro*, which could be a consequence of their cytoplasmic location where radical formation could have detrimental cellular consequences if not quenched. How B-type DyPs, prevent radical formation following compound I formation is not known, but recent work illustrating how compound I reactivity can be tuned in a wet or dry distal heme pocket may have an influence.^27,37,83^ More generally, understanding how and where functional radical sites form in DyPs and whether they can be engineered in a predicted manner has not until now received systematic attention. The work presented here, using rationally designed variants together with the simulation of a plausible mechanism, acts as an exemplar for further attempts to study radical migration and location in this peroxidase class. Given the constraints of our experimental data and hole hopping calculations, the simulation of such a complex model is illustrative rather than definitive. Nevertheless, through protein engineering of DtpAa, we learn that remarkably a relatively small change in a rate constant (equivalent to 5.7 kJ mol^−1^) brought about by the removal of a single oxygen atom (*i.e.* replacing a Tyr with a Phe in the proximal Trp/Tyr dyad) is the pivot that directs a radical from compound I to the distal side of the heme. Our findings not only have implications for understanding radical migration in peroxidases, but also provide a framework to assist future design protocols in biocatalysis applications of heme based systems, where controlling radical migration pathways to create a specific high-potential oxidising site for oxidative chemistry of an organic substrate is desirable.

## Data availability

Data supporting the conclusions of this study can be found in the main paper, with additional experimental details and results reported in the ESI.†

## Author contributions

ML, DAS and JARW conceived the study and contributed to experimental design. ML prepared all samples and together with JP measured EPR data, with assistance in the analysis by DAS. ML and MAH measured X-ray crystallography data and analysed the data. MTW and JARW developed and tested the kinetic model. JARW and DAS wrote the paper with assistance from all authors. All authors have approved the final version of the manuscript.

## Conflicts of interest

There are no conflicts to declare.

## Supplementary Material

SC-014-D3SC04453J-s001
