# Supplementary material for: New insights into controlling radical migration pathways in heme enzymes gained from the study of a dye-decolorising peroxidase
Source: Chem Sci. 2023 Oct 6;14(44):12518–34. doi: 10.1039/d3sc04453j (PMC10646903; doi:10.1039/d3sc04453j)
Supplement: SC-014-D3SC04453J-s001 [file SC-014-D3SC04453J-s001.pdf]

Electronic Supplementary Material (ESI) for Chemical Science.
This journal is © The Royal Society of Chemistry 2023

SUPPORTING INFORMATION

Title: New insights into controlling radical migration pathways in heme enzymes gained from the study of a dye-decolorising peroxidase

Marina Lučić,[1] Michael T. Wilson,[1] Jacob Pullin,[1] Michael A. Hough,[1,2] Dimitri A. Svistunenko,[1*] Jonathan A.R. Worrall[1*]

[1]School of Life Sciences, University of Essex, Wivenhoe Park, Colchester, Essex CO4 3SQ UK. [2]Diamond Light Source, Harwell Science and Innovation Campus, Didcot, Oxfordshire OX11 0DE UK.

*Corresponding author: jworrall@essex.ac.uk; svist@essex.ac.uk

EXPERIMENTAL

Site-directed mutagenesis, mutagenic primers and PCR protocol

The wild-type (WT) DtpAa DNA sequence inserted between the *NdeI* and *HindIII* restriction sites of a pET28a vector (Novagen) was used as the template for PCR reactions to create the Y345F and Y345F/F347Y constructs, with the latter used to create the Y345F/F347Y/Y389F construct. The following forward and reverse mutagenic primers were used to create the Y345F, Y345F/F347Y and Y345F/F347Y/Y389F variants of DtpAa.

F-Y345F: 5'-GCTGCGCCGCGGCTTCTCCTTCACCGACGGCA-3'

R-Y345F: 5'-TGCCGTCGGTGAAGGAGAAGCCGCGGCGCAGC-3'

F-Y345F/F347Y: 5'-GCTGCGCCGCGGCTTCTCCTATACCGACGGCA-3'

R-Y345F/F347Y: 5'-TGCCGTCGGTATAGGAGAAGCCGCGGCGCAGC-3'

F-Y389F: 5'-CGCGCTCAACGAGTTCATCCAGCACGTGG-3'

R-Y389F: 5'-CCACGTGCTGGATGAACTCGTTGAGCGCG-3'

A PCR mix consisting of the respective primers (75 ng/µl), the template (15 ng/µl), 10 mM dNTPs (Fermentas), *Pyrococcus furiosus* (*Pfu*) Turbo polymerase (Agilent), 10 x *Pfu* buffer (Agilent), 8 % DMSO and deionised H_2O to a final volume of 50 µl was prepared and subjected

to the following PCR cycle; 95 °C for 3 min; 16 cycles of 95 °C for 1 min, 58 °C for 1 min and 72 °C for 8 min; 72 °C for 15 min. All constructs generated using this PCR approach underwent Sanger sequencing (EuroFins) to verify whether they contained the desired nucleotide change(s).

Over-expression and purification of DtpAa and variants

Overnight *Escherichia coli* BL21(DE3) cultures (10 ml) containing single transformants from LB/agar plates of the respective DtpAa plasmid, supplemented with kanamycin (50 μg/ml), were used to inoculate 2 L shake flasks containing 1.4 L of high-salt LB medium (Melford Chemicals) and growth continued at 37 °C with shaking at 180 rpm. On reaching an OD_{600} of 1, the heme precursor 5-aminolevulinic acid (250 μM final concentration), an iron supplement, Fe(III) citrate (100 μM final concentration), and isopropyl β-D-thiogalactopyranoside (500 μM final concentration) were added, followed by vigorous bubbling of carbon monoxide through the liquid culture for ~30 s before sealing each shake flask with a rubber bung. Shaking continued for 16 h at 100 rpm and 30 °C, followed by centrifugation and cell lysis and loading of the supernatant to a immobilised metal-affinity chromatography column (5 ml Ni-NTA; Cytiva). Fractions containing DtpAa were concentrated using centrifugal ultrafiltration devices and applied to a preparative size-exclusion chromatography (S200; Cytiva) equilibrated in 20 mM sodium phosphate, 150 mM NaCl, pH 7.0. The purity of all DtpAa proteins was determined using SDS-PAGE, with pure fractions from the S200 column combined and stored at -20 °C until required.

Stopped-flow absorption kinetics.

Transient kinetics of the reaction of H_2O_2 with the ferric DtpAa variants was carried out using a SX20 stopped-flow spectrophotometer (Applied Photophysics) operating in a diode-array multi-wavelength mode and thermostatted to 25 °C. Variant enzyme concentrations (10 μM before mixing) were prepared in 50 mM sodium acetate, 150 mM NaCl pH 5.0 and mixed with a series of H_2O_2 concentrations ranging from 16 to 4000 μM before mixing, depending on the DtpAa variant used. Spectral transitions monitored were consistent with the presence of an intermediate species (Compound I) on going from ferric to Compound II. A sequential mechanism a $\rightarrow$ b $\rightarrow$ c was used in the Pro-K software (Applied Photophysics) to yield rate constants for Compound I (k_{obs1}) and Compound II (k_{obs2}) formation.

RESULTS

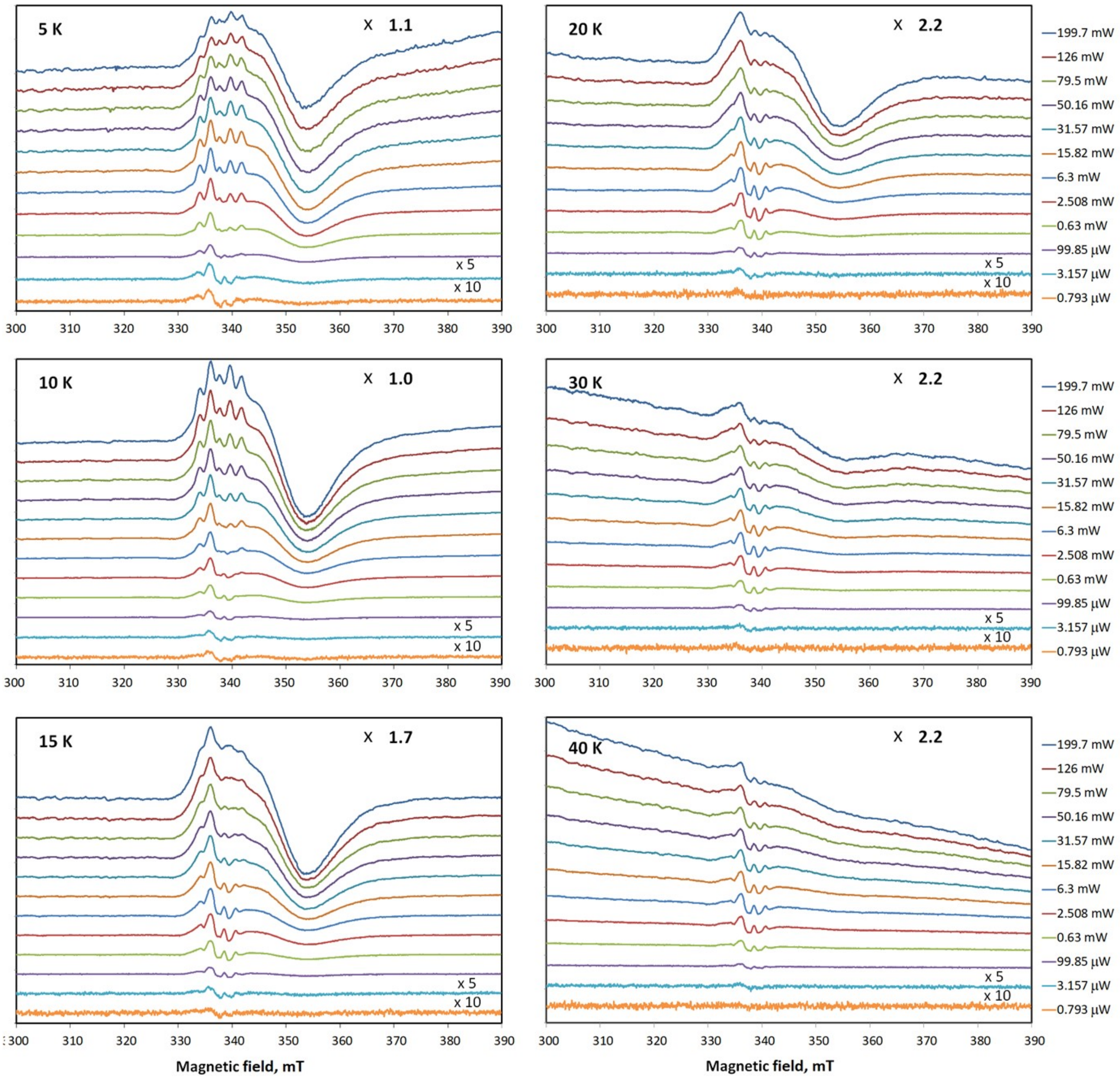

Figure S1: Microwave power / temperature saturation map of the SigA signal. Twelve MW power values (indicated on the right) have been used to measure the spectra at six different temperatures (indicated on the panes, in Kelvins). On each pane, two spectra measured at the lowest MW power values, 3.16 and 0.79 μW, are shown at magnifications 5 and 10, respectively. Different vertical scale ranges in different panes effect in different overall magnification of the spectra indicated on top right of each pane with the × sign. Instrumental parameters used to measure the spectra were as in Figure 2C.

The influence of the engineered hole hopping pathways on Compound I lifetime

Stoichiometric addition of H_2O_2 to WT ferric DtpAa, reveals changes in the absorption spectrum, consistent with the formation of a Compound II species, the lifetime of which is > 5

min.[33] On mixing WT ferric DtpAa with > 10-fold excess of H_2O_2 by stopped-flow, rapid formation of an intermediate species possessing spectral features analogous to Compound I, before decay to Compound II, as indicated in Fig. 3C, has been reported.[33] Thus, under stochiometric conditions Compound I must still form, but reduction to Compound II occurs before measurement using static absorption spectroscopy can be acquired. Stoichiometric addition of H_2O_2 to the Y345F variant, revealed identical behaviour as WT DtpAa (Fig. S2A). However, for the double variant, an absorption spectrum ~ 10 s after stoichiometric addition of H_2O_2 was recorded that displayed wavelength features to suggest the presence of a mixed Compound I and II species, which after 30 s transformed into a spectrum with pure Compound II wavelength features (Fig. S2B). For the triple variant, a Compound I spectrum was clearly observed following stochiometric H_2O_2 addition, which slowly decays over several minutes to a Compound II spectrum (Fig. S2C).

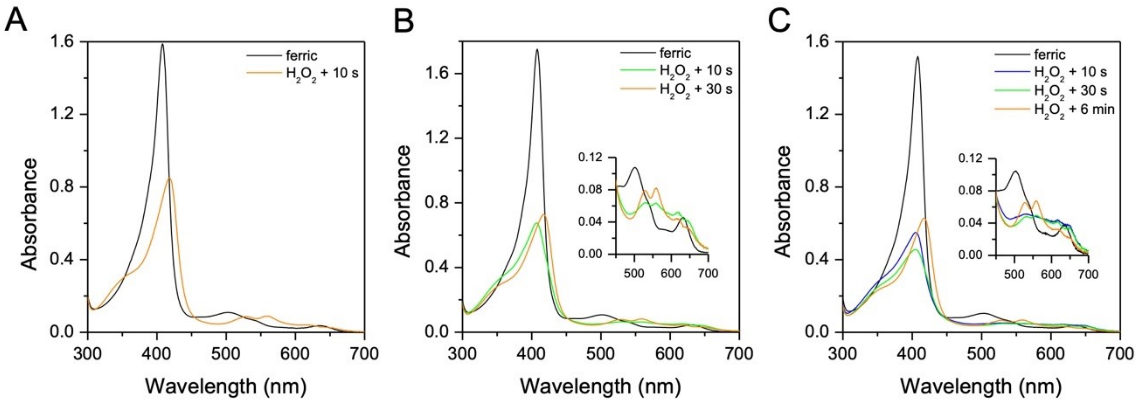

Figure S2: UV-visible absorbance spectra of DtpAa variants at pH 5.0. Spectral changes observed following stoichiometric addition of H_2O_2 to the ferric heme of the Y345F variant **A**, the Y345F/F347Y variant **B**, and the Y345F/F347Y/Y389F variant **C**. *Insets* show a zoom-in of the Q-band region.

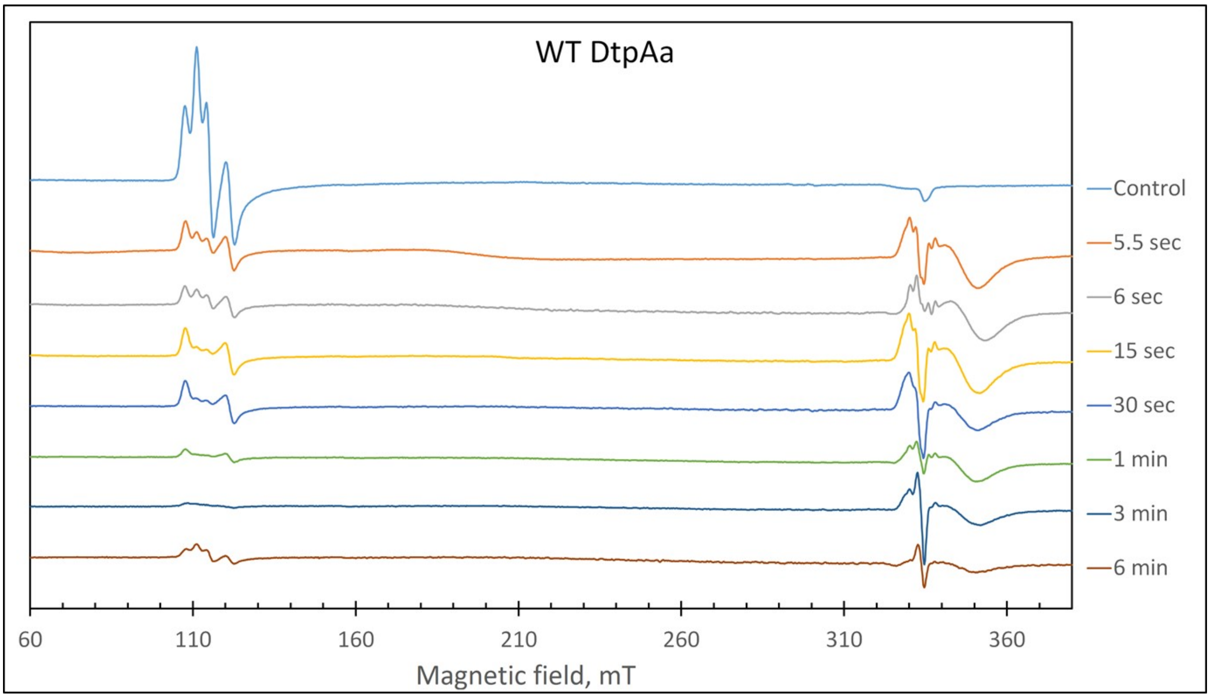

Figure S3: The EPR spectra of 40 μM WT DtpAa before (Control) and different time after mixing with 400 μM H_2O_2, pH 5. The instrumental conditions used to record the spectra were the same as in Figure 2A.

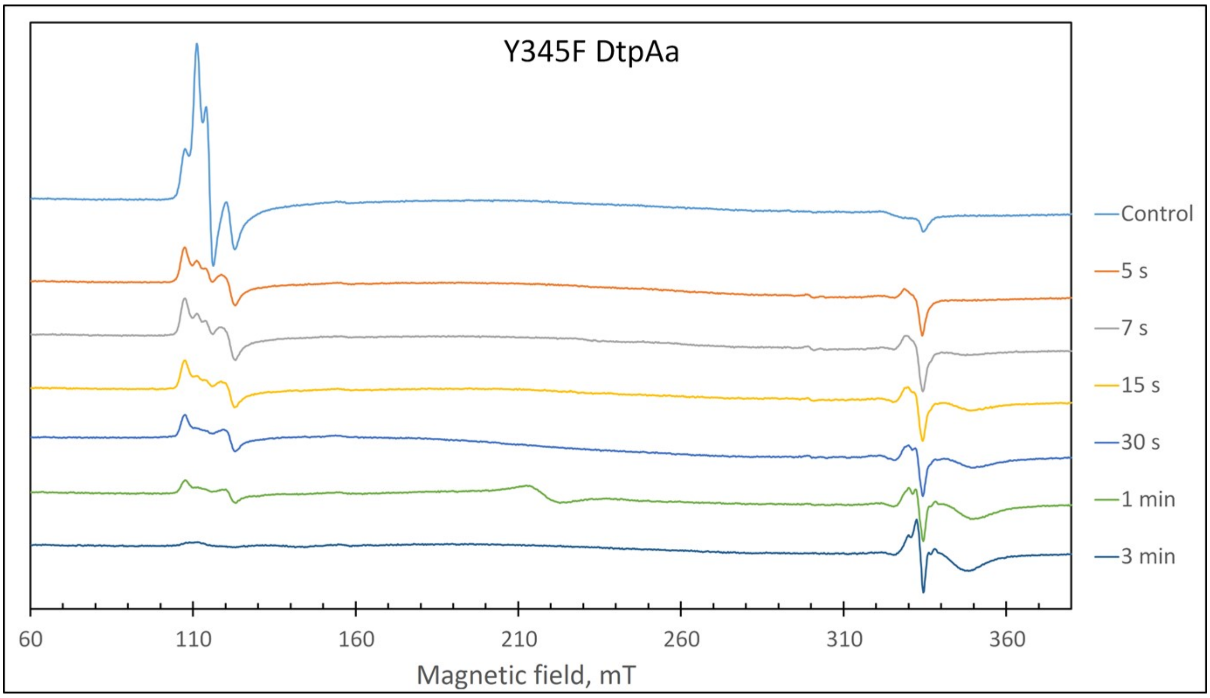

Figure S4: The EPR spectra of 40 μM Y345F DtpAa before (Control) and different time after mixing with 400 μM H_2O_2, pH 5. The instrumental conditions used to record the spectra were the same as in Figure 2A.

Table S1: Hamiltonian parameters generated by TRSSA-Y for an input of $\rho_{C1} = 0.36$ and $\theta = 56.5°$ (or the complimentary angle of 61.5°) and used to simulate EPR spectrum of the $YO^{•}$ radicals in Fig. 6F.

	x	y	z
g-values	2.00868	2.00435	2.00215
ΔH, MHz	14.73	10.65	10.42

	Principle A-values, MHz			Euler angles, degrees		
	aa	bb	cc	ϕ_1	ϕ_2	ϕ_3
$H_{\beta 1}$	18.54	15.364	15.364	27.0	5.4	26.3
$H_{\beta 2}$	14.61	11.11	11.11	-28.5	3.5	19.7
H_{C3}	25.9	8.1	20.5	-23.0	0	0
H_{C5}	25.9	8.1	20.5	23.0	0	0
H_{C2}	7.5	5.0	1.5	-40.0	0	0
H_{C6}	7.5	5.0	1.5	40.0	0	0

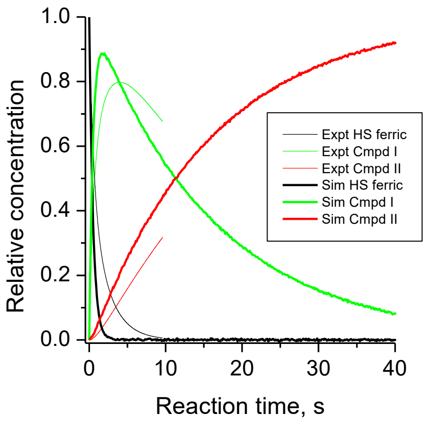

Figure S5: Optical time course simulation for the distal pathway (triple variant) using $k_5 = 2$ s^{-1} and $k_7 = 1.5$ x10^6 s^{-1}. The simulated data is overlayed with the experimental stopped-flow data for comparison.
